# Combining molecular and immunohistochemical analyses of key drivers in primary melanomas: interplay between germline and somatic variations

**DOI:** 10.18632/oncotarget.23204

**Published:** 2017-12-14

**Authors:** William Bruno, Claudia Martinuzzi, Bruna Dalmasso, Virginia Andreotti, Lorenza Pastorino, Francesco Cabiddu, Marina Gualco, Francesco Spagnolo, Alberto Ballestrero, Paola Queirolo, Federica Grillo, Luca Mastracci, Paola Ghiorzo

**Affiliations:** ^1^ Department of Internal Medicine and Medical Specialties (DiMI), University of Genoa and Ospedale Policlinico San Martino, Genoa, Italy; ^2^ Pathology Unit, Ospedale Policlinico San Martino, Genoa, Italy; ^3^ Department of Medical Oncology, Ospedale Policlinico San Martino, Genoa, Italy; ^4^ Department of Surgical and Diagnostic Sciences, Pathology Unit, University of Genoa and Ospedale Policlinico San Martino, Genoa, Italy

**Keywords:** BRAF, primary melanoma, NRAS, CDKN2A, TERT

## Abstract

Due to the high mutational somatic burden of Cutaneous Malignant Melanoma (CMM) a thorough profiling of the driver mutations and their interplay is necessary to explain the timing of tumorigenesis or for the identification of actionable genetic events. The aim of this study was to establish the mutation rate of some of the key drivers in melanoma tumorigenesis combining molecular analyses and/or immunohistochemistry in 93 primary CMMs from an Italian cohort also characterized for germline status, and to investigate an interplay between germline and somatic variants. *BRAF* mutations were present in 68% of cases, while *CDKN2A* germline mutations were found in 16 % and p16 loss in tissue was found in 63%. *TERT* promoter somatic mutations were detected in 38% of cases while the *TERT* –245T>C polymorphism was found in 51% of cases. *NRAS* mutations were found in 39% of *BRAF* negative or undetermined cases. NF1 was expressed in all cases analysed. MC1R variations were both considered as a dichotomous variable or scored. While a positive, although not significant association between *CDKN2A* germline mutations, but not *MC1R* variants, and *BRAF* somatic mutation was found, we did not observe other associations between germline and somatic events. A yet undescribed inverse correlation between *TERT* –245T>C polymorphism and the presence of *BRAF* mutation was found. It is possible to hypothesize that –245T>C polymorphism could be included in those genotypes which may influence the occurrence of BRAF mutations. Further studies are needed to investigate the role of –245T>C polymorphism as a germline predictor of *BRAF* somatic mutation status.

## INTRODUCTION

Cutaneous malignant melanoma (CMM), the most lethal form of skin cancer, also presents one of the most rapidly increasing incidences in the caucasian population [1]. A thorough profiling of the driver mutations leading melanoma progression has been a pivotal step for the identification of actionable genetic events and related targeted therapies [2]. Due to the high mutational somatic burden, CMM cannot be considered a single entity, but a set of genetically heterogeneous tumors with peculiar patterns of oncogenic mutations whose characterization could explain the molecular timing of tumorigenesis, the differences in pharmacosensitivity, the occurrence of resistance to pharmacological inhibitors and the diverse metastatic potentials [2, 3]. Further studies are still needed to appropriately interpret the mutational signatures [2, 4]. The mutations found in melanoma mostly involve the mitogen-activated protein kinase (MAPK) and the phosphoinositide 3-kinase/protein kinase B (PI3K/AKT) pathways, with *BRAF*V600E representing the most common mutation (about 60%) and mostly correlated with intermittent sun exposure [5, 6] followed by *NRAS* (28%) and *NF1*, encoding for the neurofibromin 1 protein (14%) [2, 7, 8]. Furthermore loss of expression of cyclin-dependent kinase inhibitor 2A (*CDKN2A*) gene, leading to the dysregulation of p53 or Rb activity, is observed in up to 40% of sporadic CMMs [9]. Somatic *CDKN2A* mutation was observed in 13% of melanoma cases in the TCGA analysis [7]. In familial melanoma, up to 40% of patients show germline mutations in the *CDKN2A* gene, hence, *CDKN2A* is considered the major melanoma susceptibility gene with high penetrance in the south-european area [10]. In an Italian cohort, about 10% of sporadic multiple CMM cases are *CDKN2A* mutation positive, with a further increase of the mutation rate related to the number of CMMs [11]. When *CDKN2A* founder germline mutations are present, as in the Italian population, the percentage of sporadic CMMs consecutively enrolled harboring a *CDKN2A* germline mutation is not negligible, ranging from 2 to 9% [11–13]. Variants in low to medium penetrance susceptibility gene Melanocortin-receptor type 1 (*MC1R*) [14] have been inconsistently associated with *BRAF* somatic mutation [15–20].

Recently, recurrent activating mutations in the core promoter of Telomerase Reverse Transcriptase (*TERT*), causing an increase in telomerase activity have been found in about 30–55% of primary melanomas depending on studies and up to 85% of metastatic cases and studied as an independent prognostic factor in different types of cancers including melanoma [21–28], with conflicting results regarding the association with *BRAF* mutations [29, 30]. Interestingly, the above described activating mutations in the *TERT* promoter, first described in melanoma cases, were identified following the finding of a *TERT* promoter germline mutation at nt –57 (T>G) shown to play a role in melanoma susceptibility, given its segregation in an extended melanoma family. This mutant created a new binding motif for Ets transcription factors and ternary complex factors (TCFs) near the transcription start and, in reporter gene assays, caused up to twofold increase in *TERT* transcription [22, 23]. This seminal finding stimulated the studies on the correlation and interplay between somatic and germline changes in main driver of melanomagenesis, not limited to the coding region of genes but extending to regulatory regions [29–31]. The first aim of this study was to establish the frequency of somatic mutations in some of the key driver genes of melanoma development, assessed by sequencing and/or IHC in 93 well studied [32] primary melanomas from an Italian cohort of CMMs characterized for germline status. The second aim was to correlate the germline status (*CDKN2A*/*CDK4*/*MC1R*) of patients with somatic mutations/variants or protein loss, assessed by molecular analyses and/or immunohistochemistry, in some of the key CMM drivers (*BRAF*, *NRAS*, *TERT*, NF1, p16).

## RESULTS

### Patients characteristics

Our cohort, described in a previous study [32], included 93 primary cutaneous melanomas. Of those, 59 (63%) were superficial spreading melanomas (SSM), whereas 20 (22%) were nodular melanomas (NM), and the remaining 14 (15%) were neither SSM nor NM.

Median age was 49 years, 41 were males and 52 females. The majority of melanomas were situated in the trunk (*n =* 50), followed by lower limbs (23), upper limbs (16) and head and neck (4). Median Breslow thickness was 1.6 mm. Most of the patients were stage I (52/93, 56%), followed by 30 at stage II, 7 at stage III and 3 at stage IV; one patient presented an *in situ* melanoma. Sixteen melanomas (17%) were from affected probands of melanoma families, while 74 (80%) were from sporadic cases. 12 cases (13%), 4 familial and 8 sporadic, developed multiple primary melanomas. In 3 cases (3%) information about familiarity or other primary lesions was not available.

### CDKN2A/CDK4 and MC1R germline mutations/variants

**Figure 1:**
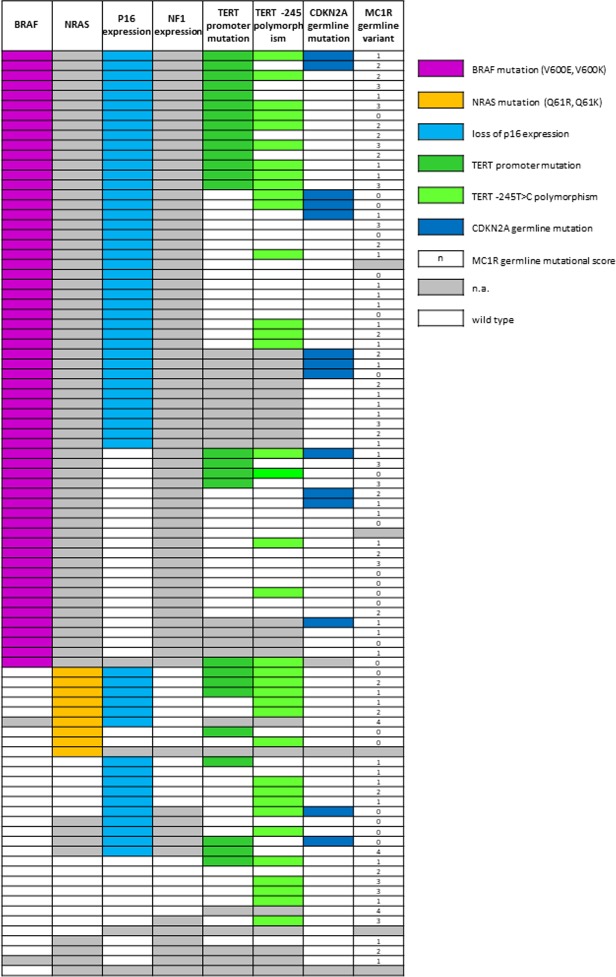
Combining results of germline and somatic analysis obtained in the study cohort N.a.: not amplified. Wild-type: for p16 and NF1 expression, white cell means that the expression of the protein is maintained; for all the genes analysed by molecular methods white cell means wild-type sequence. For *MC1R* score, wild type corresponds to the 0 score.

Germline analysis for *CDKN2A* and *CDK4* status, obtained by capillary sequencing, was available for 88/93 and 59/93 cases, respectively. All coding sequences of the *CDKN2A* gene were sequenced. We found 14 samples (16%) to be positive for mutations in the *CDKN2A* gene: 9 presented the p.G101W mutation, 2 the p.P48T, 1 the p.E27X, 1 the p.Q50R and 1 the p.A68L. We also found 2 variants of unknown significance (VUS) which were predicted as not pathogenic with in silico prediction tools (p.M1R and p.H98H which results as p.P113S on the p14 transcript). No germline mutations were found in *CDK4* exon 2. Capillary sequencing of *MC1R* gene was performed on 88 out of 93 samples and the variants retrieved were classified as indicated by Davies *et al.* [33]. We found 22 wild type samples (score 0), 33 with score 1, 18 with score 2, 12 with score 3 and 3 with score 4. In total, in our cohort 66 out of 93 samples (71%) had a *MC1R* variant of any type (“r” or “R”) (Figure 1).

### BRAF mutational status

*BRAF* mutational status was assessed as detailed in our previous study, based on the concordance among IHC, PNA clamping real-time PCR (PNA) and, eventually, capillary sequencing [32]. Four samples were previously counted as undetermined at *BRAF* status due to unsuccessful capillary sequencing or invalid PNA results. These 4 samples were re-analyzed starting from a new DNA extraction from new slides from the same blocks. Due to the concordance among two methods, one case was deemed as *BRAF* positive, one as WT and the other two were still considered *BRAF* status undetermined (grey mark in Figure 1). Following the scheme proposed, we were able to classify 62 out of 91 patients (68%) as *BRAF* mutated and 29 out of 91 as wild type (32%). Of the 62 patients classified as mutated, 60 presented the classical c.1799 T>A missense mutation (p.V600E), one presented the substitution c.1799_1800delTGinsAA (p.V600E, often reported as p.V600E2) and one the c.1798_1799delGTinsAA mutation (p.V600K).

### TERT promoter germline/somatic mutations

The analysis by capillary sequencing of the *TERT* gene promoter region was successful in 72 out of 93 tumor samples. We found 27 out of 72 samples (38%) with mutations previously described as pathological [24]. 8 (30%) showed the C>T substitution at –124 bp from the ATG start site (here called –124C>T, also indicated in literature as C228T), 17 (63%) the C>T transversion –146 bp from the ATG (here –146C>T, also described as C250T) and 2 samples (7%) both. Among the 72 amplified cases, 37 (51%) presented the –245T>C polymorphism (rs2853669) [34, 35]. In 17 out of 72 cases (24%) the variant was concomitant with *TERT* promoter mutations (Figure 1). In these patients we did not analyze germline DNA for the –57T>G mutation. This mutation, however, has never been detected in hundreds of probands from melanoma families analyzed from our cohort (data not shown).

### p16 , NRAS, NF1 mutational status and expression

**Figure 2:**
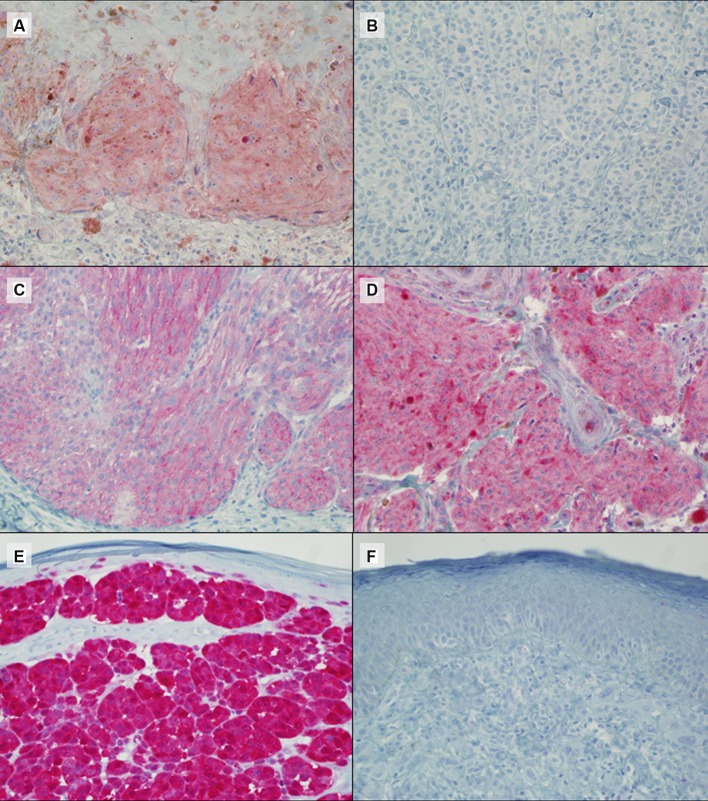
Representative IHC results for *BRAF* V600E (**A**: positive, **B**: negative), *NRAS* Q61R (**C**: positive), NF1 (**D**: positive) and p16 (**E**: positive and **F**: negative) protein expression. Magnification ×40.

The expression of p16 was investigated in 89 samples by IHC with a highly specific antibody raised against full length recombinant p16. Thirty-three samples (37%) maintained the complete or partial expression of the protein while 56 (63%) showed negative staining (Figure 2). In the *BRAF*-wt and *BRAF*-undetermined samples we investigated the mutational status of the exon 3 of *NRAS* gene, with two methods, capillary sequencing and IHC. Capillary sequencing was successful in 15/31 samples (48%) and 4 resulted mutated: 3 presented the p.Q61R mutation and 1 the p.Q61K. These results were highly concordant with results obtained by IHC although the highly specific antibody is able to recognize only the *NRAS* Q61R mutation (Figure 2) [36]. These results were then completed with the data derived from sole IHC analysis, available in 8 additional samples. In total, 9 out 23 (39%) samples were mutated in *NRAS*: 8 were identified as Q61R by IHC (three confirmed by capillary sequencing) and 1 was identified as Q61K only by capillary sequencing, as expected. For the same *BRAF*-negative and undetermined samples, we checked the loss of expression of NF1 protein with an antibody designed to recognize the N terminal portion of the NF1 protein. All the samples in which the cell content was sufficient to be evaluated (19 out of 31) showed a positive staining (Figure 2), indicating that the NF1 protein expression was not lost in the samples available for testing.

### Interplay between germline and somatic variants

**Figure 3:**
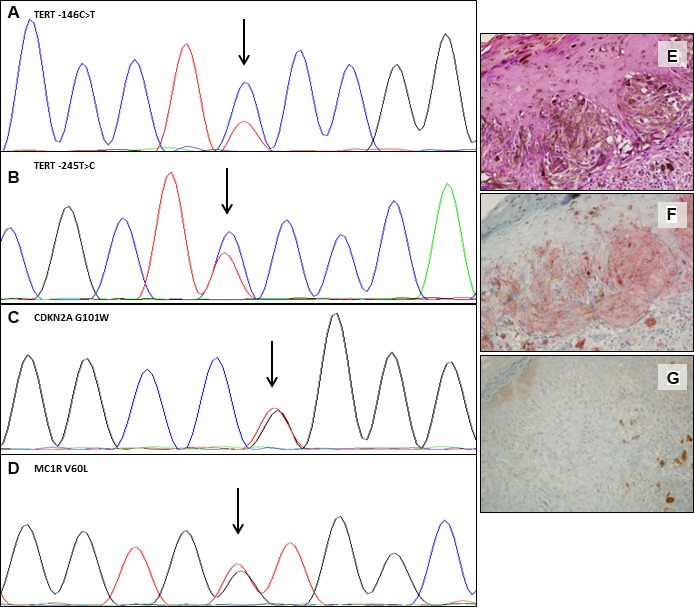
Somatic and germline mutations and variants in one representative case Electropherograms showing the *TERT* promoter –146C>T somatic mutation (**A**) and the –245T>C polymorphism (**B**); *CDKN2A* p.G101W germline mutation (**C**) and of *MC1R* p.V60L germline variant (**D**). Hematoxylin and eosin (**E**), IHC positive staining for *BRAF* V600E (**F**) and IHC showing loss of expression of p16 protein (**G**). (Magnification ×40). The variant sequence is indicated by an arrow.

Combining somatic and germline results, we observed that in 12 out of 86 (14%) cases there was co-occurrence of a somatic *BRAF* V600 mutation with a *CDKN2A* germline mutation. In line with previous studies [37], *CDKN2A* germline status (positive) showed an association with *BRAF* mutations, albeit without reaching statistical significance (OR = 3.16, *p* = 0.209; Table 1). In 45 out of 86 (52%) cases we found a somatic *BRAF* mutation combined with *MC1R* variation (any type), 18 of them (40%) included at least one “R” variant. The distribution of *MC1R* variants was not associated with *BRAF* mutations, either considering *MC1R* as a dichotomous variable (Table 1) or using the *MC1R* score as described by Davies *et al.* [33] (Kruskal–Wallis chi-squared = 0.04, degrees of freedom = 1, *p =* 0.845). *MC1R* variants were neither associated with *TERT* somatic variations nor with p16 loss (Table 1). p16 loss of expression in melanoma tissue was found in 10 out of 14 (71%) *CDKN2A* germline mutated cases, as compared to 45 out of 73 (62%) *CDKN2A* wild type cases, but this difference was not statistically significant (*p =* 0.559, Table 1). None of the *NRAS* mutated cases was *CDKN2A* germline mutation positive. Finally, only 3 out of 69 (4%) cases showed two somatic mutations (*BRAF* and *TERT* promoter) and a *CDKN2A* germline mutation (Figure 3).

**Table 1:** Association between genes and relative statistics

Gene_1	Gene_2	*N*	OR	*p*	Lower_CI	Upper_CI
BRAF	CDKN2A	86	3.16	0.209	0.63	31.23
BRAF	P16 loss	87	1.39	0.628	0.48	3.94
BRAF	TERT	72	1.09	1	0.36	3.44
BRAF	TERT -245	72	0.3	**0.025**	0.09	0.93
BRAF	TERT -245 (conservative)	44	0.15	**0.009**	0.03	0.67
BRAF	MC1R (wt/any r or R)	86	1.1	1	0.33	3.47
CDKN2A	TERT	69	1.04	1	0.19	4.96
CDKN2A	TERT -245	69	1.44	0.737	0.31	7.7
CDKN2A	MC1R (wt/any r or R)	87	0.55	0.331	0.14	2.38
CDKN2A	p16 loss	87	1.55	0.559	0.4	7.42
P16 loss	TERT	71	2.87	0.075	0.89	10.45
P16 loss	TERT -245	71	2.16	0.144	0.73	6.65
P16 loss	MC1R (wt/any r or R)	88	1.29	0.618	0.42	3.85
TERT	MC1R (wt/any r or R)	70	2.33	0.179	0.67	9.5
TERT -245	MC1R (wt/any r or R)	70	1.08	1	0.34	3.48

Abbreviations: N, OR odds ratio, CI. *TERT* -245 (conservative) = samples harboring *TERT*-245T>C polymorphism without concurrent *TERT* promoter pathogenic mutations.

### The TERT –245T>C polymorphism associates with BRAF-wt melanomas

**Figure 4:**
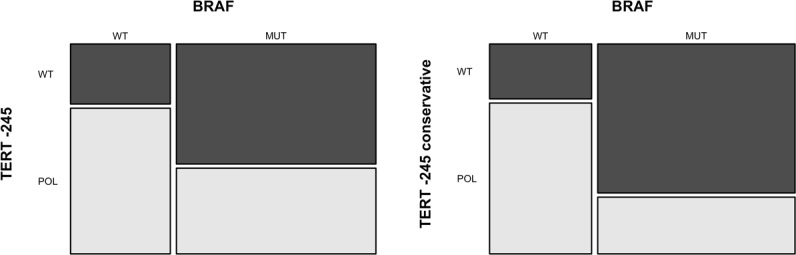
Association between the *TERT* –245T>C polymorphism and *BRAF* V600 mutation The mosaic-plot shows an inverse association between *BRAF* V600 mutation and *TERT* –245 polymorphism in primary melanoma samples (**A**). This association is stronger when only samples without concurrent *TERT* promoter pathogenic mutations are analyzed (**B**) *TERT* –245 conservative = samples harboring *TERT* –245T>C polymorphism without concurrent *TERT* promoter pathogenic mutations. MUT= mutated samples; WT = wild-type samples; POL = samples with *TERT* –245T>C polymorphism ; WT = samples without *TERT* –245T>C polymorphism.

Considering the total primary melanomas, we observed that *BRAF* mutation was concomitant in 19 out of 76 (25%) of samples with *TERT* promoter mutations and in 41 out of 62 (66%) with p16 loss of expression. In 14 out of 71 (20%) cases *BRAF* and *TERT* mutations were associated also with p16 loss of expression. The variant allele at −245 was observed in 21 out of 48 (44%) *BRAF* mutated and in 18 out of 28 (65%) *BRAF* wt. When examining the distribution of variants/mutations in our cohort, we found an association between *TERT* –245T>C polymorphism and the absence of *BRAF* pathogenic mutations (OR = 0.3, *p* = 0.025), as displayed in Figure 4A. To avoid any confounding effect by other mutations involving *TERT*, we then performed the same analysis on patients without concurrent *TERT* C228T and/or C250T mutations. In this subgroup, the inverse association between *TERT* –245T>C polymorphism and *BRAF* mutations was even stronger (OR = 0.15, *p =* 0.009, Figure 4B). In our cohort, the *TERT* –245T>C polymorphism was not associated with *TERT* promoter mutations (OR = 2.339589. *p =* 0.09542). Among the 9 primary melanoma samples which presented mutations at codon 61 of *NRAS* gene, 7 were successfully amplified for *TERT*: 4 (57%) showed mutations in *TERT* promoter and 6 (86%) presented the –245T>C polymorphism. A summary of all associations between genes and relative statistics is shown in Table 1. Due to the paucity of available data, we did not investigate the association of *NRAS* mutational status with other genes and with p16 expression.

## DISCUSSION

The aim of this study was to investigate the genetic heterogeneity of primary melanomas by means of molecular analyses and/or IHC from patients characterized for the germline status. Despite the high incidence of *CDKN2A* mutations, the number of patients in our cohort was limited, so we did not investigate prognostic features, but instead we focused on the interplay between germline and somatic variants in the key genetic drivers of cutaneous melanoma. Concerning the relationship between germline and somatic mutations, we saw positive, but not significant association between *CDKN2A* and *BRAF* mutations. These results are consistent with those of Zebary *et al.*, who found no differences in *BRAF* and *NRAS* mutation frequencies between *CDKN2A* carriers and matched sporadic cases [38]. Moreover, although a positive association between *CDKN2A* and either *BRAF* or *NRAS* mutations was initially found in a recent study by Staaf *et al.*, statistical significance was lost after adjusting for age and tumor thickness [37]. However, as opposed to recent research, *BRAF* mutations were not significantly associated with *MC1R* variants (alternatively considered as dichotomous variable or scored) and *TERT* promoter mutations or p16 loss. Moreover, we did not observe any association between *CDKN2A* status, *MC1R* and mutations in other genes or p16 loss, despite an association between TERT promoter mutations and MC1R recently found by Nagore *et al.* [39]. When investigating genetic interplay at the somatic level, we discovered that *BRAF* mutations are less frequent in patients with the *TERT* –245T>C polymorphism, especially when only considering patients without concurrent *TERT* promoter mutations. The role of *TERT* –245T>C polymorphism in cancer is debated, and research on this topic has provided controversial result. *TERT* –245T>C polymorphisms has been investigated as a poor prognostic marker in several tumors, including hepatocellular carcinoma [40] and glioblastoma [41]. However, according to Nagore *et al.*, this polymorphism is linked to improved survival in melanoma patients with *TERT* promoter mutations [28]. The hypothesis of a protective role of this particular polymorphism in cancer is supported by functional studies. In fact, Laboussiere *et al.* demonstrated that *TERT* –245T>C polymorphism downregulates *TERT* mRNA expression in gliomas, as opposed to *TERT* promoter mutations [42]. An association between the *TERT* –245T>C polymorphism and *BRAF* mutation was investigated, but not found, in a study on differentiated thyroid cancer [43], showing that *BRAF* mutation was not correlated to *TERT* –245T>C polymorphism as an additional prognostic factor. In our cohort, *BRAF* was the most frequently mutated gene, with a mutation rate of 68%. In particular, V600E was the most represented *BRAF* mutation with only 2 cases with V600K and V600E2. *NRAS* was mutated in 39% of *BRAF* WT or undetermined cases. Interestingly, *BRAF* and *NRAS* mutation rates were higher compared to previous large studies or metanalysis [7, 44] with a high concordance between capillary sequencing and IHC that strengthens the efficiency of NRAS antibody, though specific for Q61R mutation. The mutation rate of *TERT* promoter was lower if compared to a Mittle-European study [45] which found *TERT* mutations in 55% of the tumor samples with no effect on OS or association with pathologic features of aggressiveness as otherwise reported [25, 26, 24, 46, 47]. Nevertheless coexistence of a *TERT* promoter and *BRAF* mutation was detected in 19 out of 76 cases (25%), consistently with a recent Italian study [25] and occurrence of *TERT* –245T>C variant allele accounted up to 65% in *BRAF* WT cases. Our study also some limitations. As stated before, the size of our samples did not allow as to explore the relationships between germline/somatic mutations and patients’ prognosis in terms of survival and risk of relapse. Moreover, we could not analyze the association between mutations in *NRAS* and *NF1* with other germline/somatic mutations, as the information on both genes was available for a very limited number of patients. In conclusion, we studied some of the most relevant somatic mutations described in a cohort of primary CMMs characterized for germline status and we found that germinal *CDKN2A* mutations are neither associated with specific somatic mutations, nor with p16 loss of expression, confirming previous findings obtained with a combination of molecular and IHC studies [48–51]. However the mutation rate of *BRAF* was higher than previous described in studies from other populations with a peculiar inversion between the *TERT* –245T>C polymorphism and *BRAF* mutations. To the best of our knowledge this is the first study to date that describes an association between *TERT* –245T>C polymorphism and *BRAF* mutations in cutaneous melanoma. This result is of particular interest considering that *TERT* –245T>C can be frequently found as a germline polymorphism in the general population, as the minor allele frequency (MAF) is estimated to be 30%, and highest population MAF reaches 50% [Source: https://www.ensembl.org/]. Therefore, it is possible to hypothesize that specific genotypes, including the *TERT* –245T>C polymorphism, besides the debated SNPS in *MC1R* (not confirmed by the present study), may influence the occurrence of somatic *BRAF* mutations in individuals who develop cutaneous melanoma. Larger studies are needed to confirm these findings and hypothesize its role as a germline predictor of *BRAF* somatic mutation status.

## MATERIALS AND METHODS

### Case selection

A total of 100 primary melanomas were selected for a previous study on *BRAF* mutation detection [32], based on formalin-fixed, paraffin embedded (FFPE) tissue availability at the Pathology department of Policlinic Hospital, San Martino, Genoa. All melanoma patients had been referred to our center for germline testing either for diagnostic (familial and multiple melanoma cases) or research purposes (sporadic cases from a case-control study) and were part of a consecutive series of both incident and prevalent cases. All the patients signed an informed consent under local IRB approved protocols for both germline testing and other research purposes dealing with the archived melanoma tissues in the pathology department. A local database collecting information from the pathology report and tumor cell content in the examined section was designed. In this study we focused on cutaneous melanoma so we excluded seven uveal, mucosal and acral lentiginous melanomas due to the known different genetic signatures, leaving a total of 93 cases for lecular, IHC and statistical analysis. In case of patients with multiple primary melanoma only one melanoma lesion was analyzed molecularly.

### DNA extraction and capillary sequencing

Genomic DNA extraction, either from the sections or form blood withdrawal, and capillary sequencing were performed as previously described [32, 52–55]. We amplified exon 3 of *NRAS* gene, promoter region of the *TERT* gene (from –270 to –40), exon 15 of *BRAF* gene, the whole *CDKN2A* coding region (exons 1a, 1b, 2 and 3, including promoter and 3′ regions), *CDK4* exon 2 and the exon 1 of the *MC1R* gene. Sequencing reactions were repeated at least twice by independent PCR, with forward and reverse primers, and the sample was scored as being mutated when the mutation was observed both times. The same primers were used for both PCR and capillary sequencing. The in silico prediction tools we used were Poly-Phen 2, P-Mut, SIFT and Mutation taster.

### PNA clamping quantitative PCR analysis

*BRAF* V600 codon mutational status was also tested using the PNAClamp™ *BRAF* Mutation Detection Kit (Panagene, Daejeon, Korea) according to the manufacturer’s instructions, with slight modifications, as previously described [52]. The threshold cycle (Ct) was automatically calculated from the PCR amplification plots where fluorescence was plotted against the number of cycles. Delta-Ct values were calculated as the Ct values of the samples minus those of the controls. The higher delta-Ct values showed that the mutant was efficiently amplified. A cut-off value of 2.0 was used to determine the presence of mutant DNA.

### Immunohistochemistry (IHC)

For all immunohistochemical reactions, four micron-thick tissue sections were freshly cut [56], dried, deparaffinised and rehydrated. Endogenous peroxidase was blocked with 5% H2O2 for 10 minutes. Immunoreactions were performed using the automated BenchMark XT immunostainer^®^ (Ventana Medical Systems, Arizona, USA). The following antibodies were used: BRAF V600E mutation-specific antibody (Springer-Bio, clone VE1, 1:50 dilution, standard heat-based antigen retrieval was performed for 30 minutes), the NRAS antibody (LifeSpan BioSciences Inc, clone SP174, 1:50 dilution, standard heat-based antigen retrieval was performed for 60 minutes) [36], specific for Q61R mutation, NF1 antibody (Santa Cruz Biotechnology Inc., clone sc-20982, 1:20 dilution, standard heat-based antigen retrieval was performed for 60 minutes) and p16 antibody (Santa Cruz Biotechnology Inc, clone sc-56330, 1:100 dilution, standard heat-based antigen retrieval was performed for 30 minutes). The ultraVIEW Universal Alkaline Phosphatase Red Detection Kit (Ventana Medical Systems, Arizona, USA), was used. After immunostaining, slides were counterstained with haematoxylin and coverslipped. All reactions were carried out by adding positive [57] and negative controls for each run. Positive and negative controls used were chosen from metastatic samples already characterized for *BRAF* and *NRAS* molecular status for diagnostic purposes (both capillary sequencing and, for *BRAF*, PNA clamping real-time PCR, as performed by the manufacturer) whereas positive NF1 and p16 were chosen from the immunohistochemistry laboratory positive control library. All immunostained slides were simultaneously scored by two pathologists (LM and FG); disagreement was resolved by consensus. For BRAF, IHC was considered positive when cytoplasmic protein expression was scored according to the 3 categories score used by Tetzlaff *et al.* [58]. IHC evaluation for other markers was conducted as follows: NRAS (scored into 3 categories as used for BRAF, see above), NF1 (negative staining was defined as absence of any cytoplasmic staining, positive was defined as diffuse cytoplasmic positivity of any intensity) and p16 (positivity was defined using the two categories in Lade-Keller *et al.* [49].

### Statistical analysis

To assess association between two categorical variables with binary outcomes, such as mutational status of one gene or p16 loss, we performed the Fisher’s Exact test. Association between a categorical variable with binary outcome and an ordinal variable (*MC1R* score as described by Davies *et al.* [33]) was calculated using the Kruskal–Wallis test. All tests were two-sided and significance level to reject the null hypothesis was set at *p =* 0.05. Statistical analysis was carried out within the R computational environment [R Core Team (2016). R: A language and environment for statistical computing. R Foundation for Statistical Computing, Vienna, Austria [59].

## Abbreviations

CDKN2A: cyclin-dependent kinase inhibitor 2A
CMM: Cutaneous malignant melanoma
FFPE: paraffin embedded
IHC: immunohistochemistry
MAF: minor allele frequency
MAPK: mitogen-activated protein kinase
MC1R: Melanocortin-receptor type 1
PI3K/AKT: phosphoinositide 3-kinase/protein kinase B
PNA: PNA clamping real-time PCR
TCFs: ternary complex factors
TERT: Telomerase Reverse Transcriptase
