# Correction: Combining molecular and immunohistochemical analyses of key drivers in primary melanomas: interplay between germline and somatic variations

**DOI:** 10.18632/oncotarget.25684

**Published:** 2018-06-19

**Authors:** William Bruno, Claudia Martinuzzi, Bruna Dalmasso, Virginia Andreotti, Lorenza Pastorino, Francesco Cabiddu, Marina Gualco, Francesco Spagnolo, Alberto Ballestrero, Paola Queirolo, Federica Grillo, Luca Mastracci, Paola Ghiorzo

**Affiliations:** ^1^ Department of Internal Medicine and Medical Specialties (DiMI), University of Genoa and Ospedale Policlinico San Martino, Genoa, Italy; ^2^ Pathology Unit, Ospedale Policlinico San Martino, Genoa, Italy; ^3^ Department of Medical Oncology, Ospedale Policlinico San Martino, Genoa, Italy; ^4^ Department of Surgical and Diagnostic Sciences, Pathology Unit, University of Genoa and Ospedale Policlinico San Martino, Genoa, Italy

**This article has been corrected:** The proper listing of author names is given below:

William Bruno^1^, Claudia Martinuzzi^1^, Bruna Dalmasso^1^, Virginia Andreotti^1^, Lorenza Pastorino^1^, Francesco Cabiddu^2^, Marina Gualco^2^, Francesco Spagnolo^3^, Alberto Ballestrero^1^, Paola Queirolo^3^, Federica Grillo^4^,^*^, Luca Mastracci^4^,^*^ and Paola Ghiorzo^1^,^*^

Original article: Oncotarget. 2018; 9:5691-5702. https://doi.org/10.18632/oncotarget.23204

